# Neurocognitive and Quality of Life Improvements Associated With Aerobic Training for Individuals With Persistent Symptoms After Mild Traumatic Brain Injury: Secondary Outcome Analysis of a Pilot Randomized Clinical Trial

**DOI:** 10.3389/fneur.2019.01002

**Published:** 2019-09-18

**Authors:** Emily Gladstone, Megan E. Narad, Fadhil Hussain, Catherine C. Quatman-Yates, Jason Hugentobler, Shari L. Wade, Paul J. Gubanich, Brad G. Kurowski

**Affiliations:** ^1^Department of Physical Medicine and Rehabilitation, MetroHealth and Case Western Reserve College of Medicine, Cleveland, OH, United States; ^2^Division of Behavioral Medicine and Clinical Psychology, Department of Pediatrics, Cincinnati Children's Hospital Medical Center, University of Cincinnati College of Medicine, Cincinnati, OH, United States; ^3^College of Allied Health Sciences, University of Cincinnati, Cincinnati, OH, United States; ^4^Division of Occupational Therapy and Physical Therapy, Department of Physical Therapy, Cincinnati Children's Hospital Medical Center, The Ohio State University, Columbus, OH, United States; ^5^Division of Physical Therapy, Sports Medicine Research Institute, and Chronic Brain Injury Program, Columbus, OH, United States; ^6^Division of Occupational Therapy and Physical Therapy, Cincinnati Children's Hospital Medical Center, Cincinnati, OH, United States; ^7^Division of Pediatric Rehabilitation Medicine, Department of Pediatrics, Cincinnati Children's Hospital Medical Center, University of Cincinnati College of Medicine, Cincinnati, OH, United States; ^8^Division of Sports Medicine, Department of Pediatrics, Cincinnati Children's Hospital Medical Center, University College of Medicine, Cincinnati, OH, United States; ^9^Department of Internal Medicine, University College of Medicine, Cincinnati, OH, United States; ^10^Division of Pediatric Rehabilitation Medicine, Departments of Pediatrics and Neurology and Rehabilitation Medicine, Cincinnati Children's Hospital Medical Center, University of Cincinnati College of Medicine, Cincinnati, OH, United States

**Keywords:** mTBI (mild traumatic brain injury), aerobic training, neurocognitive, quality of life, pediatrics

## Abstract

**Objective:** To report secondary neurocognitive and quality of life outcomes for a pilot randomized clinical trial (RCT) of aerobic training for management of prolonged symptoms after a mild traumatic brain injury (mTBI) in adolescents.

**Setting:** Outpatient research setting.

**Participants:** Thirty adolescents between the ages of 12 and 17 years who sustained a mTBI and had between 4 and 16 weeks of persistent post-concussive symptoms.

**Design:** Secondary outcome analysis of a partially masked RCT of sub-symptom exacerbation aerobic training compared with a full-body stretching program highlighting cognitive and quality of life outcomes.

**Main Measures:** The secondary outcomes assessed included neurocognitive changes in fluid and crystallized age-adjusted cognition using the National Institutes of Health (NIH) toolbox and self and parent-reported total quality of life using the Pediatric Quality of Life Inventory.

**Results:** Twenty-two percent of eligible participants enrolled in the trial. General linear models did not reveal statistically significant differences between groups. Within group analyses using paired *t*-tests demonstrated improvement in age-adjusted fluid cognition [*t*_(13)_ = 3.39, *p* = 0.005, Cohen's *d* = 0.61] and crystallized cognition [*t*_(13)_ = 2.63, *p* = 0.02, Cohen's *d* = 0.70] within the aerobic training group but no significant improvement within the stretching group. Paired *t*-tests demonstrated significant improvement in both self-reported and parent-reported total quality of life measures in the aerobic training group [self-report *t*_(13)_ = 3.51, *p* = 0.004, Cohen's *d* = 0.94; parent-report *t*_(13)_ = 6.5, *p* < 0.0001, Cohen's *d* = 1.80] and the stretching group [self-report *t*_(14)_ = 4.20, *p* = 0.0009, Cohen's *d* = 1.08; parent-report *t*_(14)_ = 4.06, *p* = 0.0012, Cohen's *d* = 1.045].

**Conclusion:** Quality of life improved significantly in both the aerobic exercise and stretching groups; however, this study suggests that only sub-symptom exacerbation aerobic training was potentially beneficial for neurocognitive recovery, particularly the fluid cognition subset in the NIH Toolbox. Limited sample size and variation in outcomes measures limited ability to detect between group differences. Future research should focus on developing larger studies to determine optimal timing post-injury and intensity of active rehabilitation to facilitate neurocognitive recovery and improve quality of life after mTBI.

**Clinical Trial Registration:** www.ClinicalTrials.gov, identifier: NCT02035579.

## Introduction

Mild traumatic brain injury (mTBI) is a significant cause of morbidity in adolescents, however, little research exists on how best to aid neurocognitive recovery and maximize quality of life for these children. Children and young adults are estimated to have over one-million emergency department visits annually in the United States (1). While physical symptoms such as headache, nausea, and blurry vision are common after mTBI, neurocognitive deficits also need to be addressed to ensure the highest level of recovery. Children often present with neurocognitive changes including poor concentration, decreased processing speed, and slowed reaction time after mTBI (2–4). These symptoms can make return to school more difficult and every day functioning challenging for the children and their families. After mTBI, the majority of children have complete resolution of their symptoms in the first few weeks, however, a small portion continue to face recovery challenges, with ~12% of children showing persistent symptoms 3 months post-injury (5). Studies indicate a history of multiple mTBIs and pre-existing psychiatric conditions put children at risk for prolonged recovery and potentially, a reduction in quality of life related to persistent problems (6).

Mild traumatic brain injury causes dysregulation of cerebral blood flow and neurochemical changes in the brain resulting in a metabolic and energy imbalance that can persist months post-injury (4, 7). Evidence supports that these physiologic alterations are responsible for mTBI symptoms including mental fogginess, fatigue, mood changes, and emotional disturbances (8). These symptoms may contribute to known neurocognitive changes after mTBI including altered attention, processing time, and working memory (3, 8).

Currently, there is no definitive treatment for persistent symptoms after mTBI. Aerobic exercise is a potentially promising intervention as it is believed to improve cerebral blood flow and neurometabolic physiology in the brain (9, 10). Aerobic exercise is felt to improve physiologic brain dysfunction and consequently have a positive impact on neurocognitive symptoms (9, 10). Aerobic exercise programs have emerged as a safe and feasible rehabilitation strategy for patients with mTBI (11–15).

Multiple studies focusing on mTBI recovery provide preliminary evidence for the efficacy of aerobic exercise in promoting symptom improvement (11–15). A recent systematic review identified a variety of positive outcomes related to exercise training after mTBI (12). Exercise has been associated with reduced symptoms, faster return to full function, reduced days of recovery, and improved reaction times (11, 13–15). However, these prior findings were limited with regard to other outcome measures, including neurocognitive and neuropsychological outcomes, and there is a paucity of RCTs characterizing how exercise may specifically result in improvements in such outcomes. Animal models evaluating aerobic exercise have demonstrated positive changes in gene plasticity of the hippocampus, which is responsible for memory (16), raising the possibility of improvements on neurocognitive testing in adolescents who engage in aerobic exercise training. It is critical to evaluate the range of potential advantages of aerobic exercise as a treatment, especially in the adolescent population, for whom a non-invasive and non-pharmacologic option may be particularly beneficial.

In the original report of this exploratory randomized clinical trial (RCT), participants in both the aerobic and stretching groups demonstrated a downward trend in Post-Concussion Symptom Inventory (PCSI) ratings, with the aerobic training group showing a greater decrease in PCSI ratings than the stretching group (11), indicating that a sub-symptom exacerbation aerobic exercise program may be beneficial to patients with post-concussive symptoms compared to a full-body stretching program. Since the stretching group showed improvement as well, minimal activity may even be beneficial in the recovery process.

The goal of this paper was to characterize the effect of aerobic training on the secondary outcomes of neurocognitive functioning and quality of life. Neurocognitive outcomes were assessed using the fluid and crystallized cognitive subsets of the NIH Toolbox (17, 18). Fluid cognition includes episodic and working memory, processing speed, attention and executive function while crystallized cognition encompasses language (18). We hypothesize that fluid cognitive function would improve with aerobic exercise as it is often affected after mTBI (3, 9). Furthermore, crystallized cognition, which is considered a fixed measure, is not expected to change with intervention. Additionally, we hypothesized that aerobic training would also be associated with improvements in quality of life.

## Methods

### Design

We conducted an exploratory RCT to determine the benefits of a 6-week, sub-symptom exacerbation aerobic training compared to a full-body stretching intervention in adolescents with persistent symptoms after injury. This clinical trial was registered through clinical trials and the registration number is NCT02035579. Randomization was stratified by age (12–14 and 15–17 years) and sex. The study was single blinded; evaluators were unaware of group assignment (11).

### Participants

As described previously (11), adolescents between the ages of 12 and 17 years were recruited from outpatient clinics, emergency departments, and communities throughout the Cincinnati, Ohio area between September 1, 2013 and February 1, 2015. Individuals were considered for participation if they had sustained an mTBI, experienced between 4 and 16 weeks of persistent symptoms, and endorsed that these symptoms were exacerbated with physical activity. Table 1 lists exclusion criteria that were applied.

**Table 1:** Exclusion criteria.

1. Unable to speak and or read English
2. Evidence of more severe TBI
3. Preexisting neurological impairment
4. Cognitive disorders
5. History of psychological diagnosis
6. Developmental delay
7. Genetic disorders
8. Metabolic disorder
9. Cognitive disorders
10. Hematologic disorders
11. Cancer
12. Neck pain
13. Pre-injury diagnosis of ADHD
- Requiring 2+ medications
- Medication changes in last 1 month
14. Current participation in other therapy
15. History of cardiovascular problems
16. Recent/upcoming medication dose changes
- Beta-blockers
- Antidepressants
- Antianxiety medications
- ADHD medications
- Prophylactic headache medications
- Mood behavioral medications

**Figure 1:**
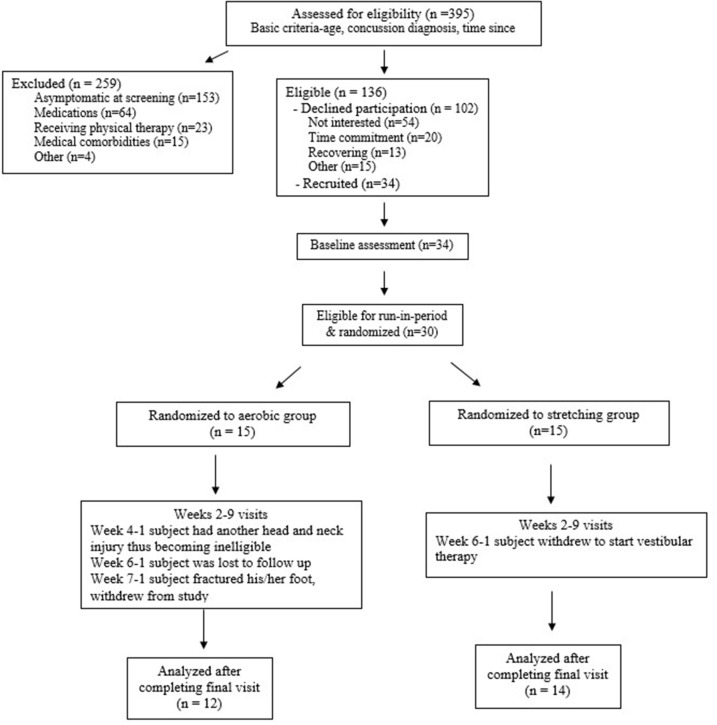
CONSORT flow chart [adapted from Kurowski et al. (11)].

As reported previously (11), a total of 395 individuals were evaluated for eligibility and 136 were deemed eligible (Figure 1). Of the eligible participants, 102 declined participation due to lack of interest, time commitment, ongoing recovery, and “other” reasons. Thirty-four completed a baseline assessment. At the baseline assessment, four individuals were excluded because of medication usage or lack of symptoms during the biking protocol. A total of 30 participants were randomized. All participants provided written informed consent and assent in accordance with good clinical practices and local IRB standards. Written parental consent was obtained for all participants under the age of 16.

### Study Procedure

At the baseline visit (week 0), participants were evaluated for eligibility and an aerobic bike test was conducted. Participants began biking at a speed consistent with a Borg rate of perceived exertion (RPE) of 11 (fairly light), with the bikes (Exerpeutic upright exercise bike) set at resistance at level 2, for 5 min, then increased RPE by 1 at 5-min intervals for 30 min (max intensity of 16) or until they experienced symptoms. Participants unable to complete at least 2 min of cycling before experiencing symptoms and participants able to complete the full 30 min test without experiencing symptoms were excluded from further participation in the trial. Participants who were not excluded moved to a run-in period, allowing an opportunity to monitor for any changes in symptoms that may occur as part of natural recovery (Figure 1).

At the week 1 visit, participants were again evaluated for eligibility then randomized into either the sub symptom exacerbation aerobic training group or the full body stretching group (11). The aerobic training group repeated the aerobic cycling test that was performed at the baseline assessment in order to create a customized home exercise program. Participants were given the same model exercise bike to use at home for the duration of the study and were asked to complete their individually tailored cycling program 5–6 days per week at 80% of the duration that exacerbated symptoms during study visits. The cycling program was repeated at each of the following six visits and based on the results, the home exercise program was adjusted for each participant in the aerobic training group. Participants returned the bikes at study completion. Participants in the stretching group were instructed on a full-body stretching program to be completed at home 5–6 days per week. The stretching group reviewed the program at weekly intervals and received a new group of stretches every 2 weeks, targeting a variety of upper extremity, lower extremity, and trunk muscles (11).

Participants in each arm of the study were asked to complete at least 6 weeks of training. They were considered fully recovered and transitioned to a run-out period if they were able to complete the cycling test without symptom exacerbation. Those who did not return to their baseline after 6-weeks of training remained in the program for up to two additional weeks prior to moving to the post-intervention run-out period (11).

### Adherence and Adverse Events

Adherence and adverse events for this study were reported previously (11). Participants in the stretching group reported completing sessions more times per week than the aerobic exercise group, mean number of times per week were 5.85 (1.37) and 4.42 (1.95) (*p* < 0.0001), respectively (11). Adverse events encountered during the study were unrelated to the study protocol itself, for example one subject fractured his/her foot and another had a new head and neck injury (11).

### Outcome Measures

Both the NIH Toolbox and PedsQL (self-report and parent proxy forms) were administered at baseline and at the end of the intervention. The NIH Toolbox is a multidimensional set of measures that can be used to assess cognitive, sensory, motor, and emotional function in individuals ages 3–85 years (17). The Toolbox has been validated and normed in a broad sample of the US population. The NIH Toolbox Cognition Battery was used to assess global cognition (17). It consists of tests of executive function, attention, episodic memory, language, processing speed, and working memory (17). This battery yields the following summary scores, in addition to individual measure scores: Cognitive Functioning Composite Score, Fluid Cognition Composite Score, and Crystallized Cognition Composite Score. Composite scores (age-adjusted crystallized cognition, age adjusted fluid cognition, and age adjusted total cognition scores) were used as dependent variables.

The pediatric Quality of Life Inventory (PedsQL) generic core was used to assess quality of life. The PedsQL is composed of 23 items that measure physical, emotional, social, and school functioning (19–21). Self-report forms are validated for children 5–18 years and parent-report forms have been developed for children 2–18 years. The PedsQL has been used in pediatric TBI as a measure of quality of life (22–25). PedsQL total score was used as the dependent variable in analyses.

Other outcome variables and results from this RCT have previously been published, including findings that aerobic exercise training may result in faster symptom recovery (11) and improved structural connectivity in brain networks (26).

### Sample Size

In the primary outcome paper (11), we determined that 15 participants would be needed per group to detect an effect size of 1.25 at an alpha of 0.05, power of 0.9 and a 10% drop out rate (11). Effect sizes of 0.2, 0.5, and 0.8 are consider small, medium, and large, respectively (27). Power and sample size calculations were based on symptom recovery trajectory.

### Analysis

General linear models were used initially to examine the effect of group (aerobic vs. stretching) status on post-intervention scores, controlling for baseline scores. No significant group effects were noted in any of the post-intervention outcomes. Due to the pilot/exploratory nature of this study, separate paired samples *t*-tests were used to explore pre-post differences within each group, and Cohen's *d* was used to characterize the magnitude of change within each group. The Shapiro-Wilk (28) test indicates that data are unlikely to violate normality for all outcomes evaluated (*p* > 0.05), except for the post-intervention self-report PedsQL (*p* = 0.04) and parent-report PedsQL (*p* = 0.01) in the aerobic group (see Supplemental Table 1).

## Results

There were no between group differences noted for participant race, sex, age, primary caregiver level of education, household income, prior history of concussion, and time since injury. The full body stretching group was more likely to have a non-sports related mechanism of injury but both groups had the same number of participants in organized sport (Table 2). In the aerobic training group, six out of 12 did not return to baseline and required a further 2 weeks of training. Individuals in the aerobic group that returned to baseline earlier were similar to individuals that did not return to baseline with respect to age, sex, race, time since injury, and initial symptom ratings (11).

**Table 2:** Comparison of baseline data between intervention and comparison groups [adapted from Kurowski et. al. (11)].

	**Cycling** **(*n* = 15)**	**Stretching** **(*n* = 15)**	***P*-value**
Age at enrollment, mean (SD), *y*	15.22 (1.37)	15.50 (1.80)	0.64
Time since injury, mean (SD), *d*	52.30 (19.93)	55.95 (22.16)	0.64
Sex (males), *n*	5	8	27
Race (non-white), *n*	2	2	1.00
Primary caregiver education (with bachelor degree or higher), *n*	9	7	0.46
Income ($70,000 and above annual income), *n*	9	9^a^	0.81
Mechanism of injury (sports-related), *n*	6	12	0.03
Number reporting typical participation in an organized sport prior to injury, *n*	13	13	1.00
History of two or more concussions (including injury related to this study), *n*	10	6	0.14

a *The value is based on n = 14, primary caregiver unavailable or declined to provide information*.

General linear models did not reveal statistically significant effect of treatment group (aerobic and stretching groups) on any of the neurocognitive or quality of life measures. However, paired *t*-test models demonstrated significant pre-post differences in the fluid and crystallized age-adjusted test measures from the NIH Toolbox (Table 3) within the aerobic training group but not the stretching group. The aerobic training group demonstrated a significant pre-post increase in age-adjusted fluid cognition [*t*_(13)_ = 3.39, *p* = 0.005, Cohen's *d* = 0.611] and age-adjusted crystallized cognition scores [*t*_(13)_ = 2.63, *p* = 0.02, Cohen's *d* = 0.704]. The stretching group failed to demonstrate significant pre-post changes in fluid cognition [*t*_(13)_ = 1.08, *p* = 0.30, Cohen's *d* = 0.338] or crystallized cognition scores [t_(14)_ = 1.79, *p* = 0.09, Cohen's *d* = 0.425]. A significant pre-post increase in the age-adjusted total cognition score was noted for both the aerobic training [*t*_(13)_ = 5.13, *p* = 0.0002, Cohen's *d* = 1.370] and stretching groups [*t*_(13)_ = 3.10, *p* = 0.01, Cohen's *d* = 0.82].

**Table 3:** Neurocognitive and quality of life scores.

**Measure**	**Aerobic exercise group**					**Stretching group**				
	**Visit1**	**Visit2**	***t*-test**	***p*-value**	**Cohen's d**	**Visit1**	**Visit2**	***t-*test**	***p*-value**	**Cohen's *d***
Fluid mean (SD)	88.98 (23.66)	107.36 (17.97)	*t*_(13)_ = 3.39	*p* = 0.005	0.611	99.38 (22.78)	105.28 (19.31)	*t*_(13)_ = 1.08	*p* = 0.30	0.338
Crystallized mean (SD)	101.29 (1.25)	107.60 (13.61)	*t*_(13)_ = 2.63	*p* = 0.02	0.704	107.27 (11.64)	110.48 (14.50)	*t*_(14)_ = 1.79	*p* = 0.09	0.463
Self-report PedsQL Mean (SD)	72.25 (14.68)	85.64 (12.09)	*t*_(13)_ = 3.51	*p* = 0.004	0.9375	71.09 (14.55)	81.64 (13.68)	*t*_(14)_ = 4.20	*p* = 0.0009	1.084
Parent-report PedsQL Mean (SD)	60.29 (12.25)	86.94 (12.46)	*t*_(12)_ = 6.50	*p* < 0.0001	1.802	61.59 (16.37)	80.00 (15.85)	*t*_(14)_ = 4.06	*p* = 0.0012	1.048

Individual subgroups of fluid cognition in the aerobic exercise group showed statistically significant improvement on all subtests including attention, reaction time, cognitive flexibility, episodic memory, working memory, and processing speed tested by flanker inhibition [*t*_(13)_ = 3.90, *p* = 0.0018, Cohen's *d* = 1.040], dimensional change card sorting [*t*_(13)_ = 3.16, *p* = 0.01, Cohen's *d* = 0.844], picture sequencing [*t*_(13)_ = 3.09, *p* = 0.0085, Cohen's *d* = 0.827], list sorting [*t*_(13)_ = 3.07, *p* = 0.0090, Cohen's *d* = 0.820] and pattern comparison [*t*_(13)_ = 4.0, *p* = 0.0015, Cohen's *d* = 1.070].

Paired *t*-tests also revealed substantial within group differences with pediatric quality of life total score (Table 3). Significant pre-post increases on the PedsQL Total score were noted for both the aerobic [*t*_(13)_ = 3.51, *p* = 0.004, Cohen's *d* = 0.938] and the stretching [*t*_(13)_ = 4.2, *p* = 0.0009, Cohen's *d* = 1.084] groups. A similar pattern was noted for the equivalent parent-reported PedsQL measure with significant increases in Total PedsQL score in the aerobic [t_(12)_ = 6.50, *p* < 0.0001, Cohen's *d* = 1.802] and stretching [t_(12)_ = 4.06, *p* = 0.0012, Cohen's *d* = 1.048] groups.

## Discussion

Secondary findings of this exploratory RCT suggest sub-symptom exacerbation aerobic training is potentially beneficial (or at least not detrimental) for neurocognitive recovery and quality of life. This is consistent with the shift in current literature to support active rehabilitation programs in mTBI recovery instead of the classically prescribed rest (11, 12, 15, 29). Overall, age-adjusted scores in the fluid cognition category showed within-group improvement for the aerobic exercise participants but not those receiving the stretching intervention. These results are generally consistent with findings in a meta-analysis demonstrating improvement in reaction time scores, a fluid measure, with exercise after mTBI (12). Another recent study has also demonstrated improvement in quality of life when children with mTBI participate in an active exercise rehabilitation program (30). Understanding the presumed relationship between aerobic exercise and neurocognition improvements in the mTBI population requires a deeper look at a cellular and molecular level. Aerobic training has been shown to impact neuroplasticity through variety of mechanisms (31). With exercise, nerve growth factor (NGF) increases in the brain, leading to neurogenesis in the dentate gyrus of the hippocampus, which is ultimately responsible for learning and memory. Increased cerebral blood flow with aerobic exercise is thought to be the result of angiogenesis related to increased vascular endothelial growth factor (VEGF). Both of these mechanisms potentially may help explain why the improvement in fluid cognition were most notable in the aerobic exercise group. Timing is a key consideration when recommending aerobic exercise following mTBI. We studied adolescents starting aerobic exercise at least 4 weeks post-injury and were able to see significant improvements in fluid cognition. Recent studies demonstrated that earlier initiation of such protocols potentially leads to faster resolution of symptoms and earlier return to sport and school (14, 15), specifically initiating activity within 7 days may lead to a decrease in symptoms at a month (32). Our results may have shown even more dramatic improvement if aerobic exercise was initiated sooner in the recovery course. Determining the optimal timing of initiation and intensity of exercise that is optimal for each individual in their recovery course is critical needed for the field.

The ideal exercise intensity that should be utilized for managing recovery after TBI is unclear. The study described in this paper utilized a moderate intensity biking protocol which has been consistently demonstrated to be beneficial. Majerske et al. found that moderate intensity exercise after mTBI resulted in better outcomes on computerized neurocognitive testing when compared with high or low intensity activities (33). Another study demonstrated that strenuous exercise protocols are associated with improved post-concussive symptoms; however, neurocognitive recovery declined from day 2 to day 10 after initiation of the protocol (13). Studies in healthy children, adolescents and adults have shown that strenuous activity immediately to up to 3 h before testing can negatively affect cognitive assessment (34, 35). However, in other healthy and neurologic populations, high-intensity exercise seems to have a greater impact on brain plasticity and brain health (36–44). There is a critical need to continue to develop improved evidence to inform exercise prescription for children and adolescents after mTBI.

Our results supported the hypothesis that exercise may have a greater influence on fluid cognition, such as working memory, processing speed, and executive functioning, than crystallized cognition after mTBI. This finding is in agreement with other work that has demonstrated that fluid abilities are most affected by mTBI (3). Although learning effects typically are accounted for by the NIH Toolbox for crystallized cognition (17), due to the close, repetitive testing performed in this study, some learning effects may be present (45). Therefore, learning effects could account for the subtle improvements in crystallized cognition seen in both the aerobic and stretching groups. For subsets of fluid cognition, the largest effect size was seen in the aerobic training group for the processing speed tests. Because both groups' scores at the final visit in our study were above the average fluid cognition subset score, the possibility of a learning effect should be considered.

We must also consider the fact that a truly “inactive” control group was not utilized since the stretching group still received an active, albeit minimal activity, intervention. Therefore, the improvement seen with the aerobic exercise may be more striking if compared to a true physical rest placebo (46). One study examined the effect of strict rest for 5 days following concussion and found higher report of symptoms and slower recovery in those with strict rest compared to those with 1–2 days of rest and progressive, gradual resumption of activity (46).

This secondary analysis also evaluated overall quality of life and demonstrated improvement in overall quality of life for both the self- and parent-reported PedsQL in the aerobic and stretching groups. This suggests that even a small amount of activity may have a positive influence on adolescent quality of life after mTBI in the setting of persistent post-injury symptoms. Alternately, since both protocol groups improved, these findings may be an artifact of natural recovery.

## Limitations

The generalizability of this exploratory trial is limited by a variety of factors including sample size, high non-participation rate during selection process, exclusion of patients with neck and cervicogenic symptoms, and limited racial and socioeconomic diversity (11). Age and sex characteristics were similar between groups; however, due to the small sample size, subgroups analyses based on these factors is unwarranted; future larger studies are needed to better understand the association of age and sex with response to exercise. Additionally, this study focused on adolescences; therefore, extrapolation of findings to younger populations should be done with caution and future studies should potentially focus on this younger age range. Also, due to the small sample size, the study is at risk for non-normal distribution of data; therefore, interpretation of findings should be done with caution. Overall, there is a need for future studies with larger sample sizes to confirm the results of this study and characterize the association of an array of individual and injury factors on response to the intervention and outcomes to develop better precision medicine approaches. The full body stretching group was more likely to have a non-sports related mechanism of injury; however, reports of participation in sports was similar between groups. Higher baseline fluid cognition scores were noted in the stretching group compared with the aerobic group at initial testing. This may be due to an unknown characteristic which was not accounted for during study randomization. The higher baseline would have made it more difficult for the stretching group to improve compared to the aerobic group.

It is also important to note that fluid and crystallized cognition scores for both groups generally fell within the normal range. The average crystallized cognition scores before and after the protocols were above average for age. In addition, the baseline fluid cognition scores were below average and lower in the aerobic group compared to the stretching group which may have contributed to the significant changes found in this cognition subset. Another consideration is a ceiling effect may potentially be present since the post-intervention scores were above average in both fluid and crystallized cognition. The practice effect is another limitation of the study. It has been demonstrated that practice effects unlikely exist for crystallized cognition but might be important for fluid cognition (45). Therefore, it is difficult to say with absolute certainty how much of the improvement in fluid cognition is related to intervention, expected recovery, and practice effects.

Outcome reporting may have been biased since the structure of the study did not allow for double blinding. Reporting bias should be considered in regards to adherence as this was assessed by self-reported and parent-report only. It is possible that participants may have participated in activities outside of the study protocol that were unaccounted for and could have influenced the results.

## Conclusion

Secondary findings from this exploratory RCT support that sub-symptom exacerbation aerobic training may potentially have positive effects on the neurocognitive recovery of fluid cognitive abilities such as working memory and executive function skills in adolescents with persistent symptoms after mTBI. Data also suggests that improvements in quality of life may be seen with both stretching and aerobic exercise protocols in this population. There is a critical need for continued development of evidence-based treatments for management of mTBI. Future studies should aim to determine optimal intensity and timing of physical activity after mTBI and how this can positively influence neurocognitive outcomes, quality of life, and global functioning.

## Data Availability

The datasets generated for this study are available on request to the corresponding author.

## Ethics Statement

This study was carried out in accordance with the recommendations of Cincinnati Children's Institutional Review Board (IRB) with written informed consent from all subjects. All subjects gave written informed consent in accordance with the Declaration of Helsinki. The protocol was approved by the Cincinnati Children's IRB.

## Author Contributions

Each author has made substantial contributions to study design, implementation, analysis, and/or write-up. All authors accept responsibility for reported research, and all authors have participated in the concept and design, analysis and interpretation of data, drafting or revising of the manuscript, and have approved the manuscript as submitted.

### Conflict of Interest Statement

The authors declare that the research was conducted in the absence of any commercial or financial relationships that could be construed as a potential conflict of interest.
