# Supplementary material for: Neurocognitive and Quality of Life Improvements Associated With Aerobic Training for Individuals With Persistent Symptoms After Mild Traumatic Brain Injury: Secondary Outcome Analysis of a Pilot Randomized Clinical Trial
Source: Front Neurol. 2019 Sep 18;10:1002. doi: 10.3389/fneur.2019.01002 (PMC6759771; doi:10.3389/fneur.2019.01002)
Supplement: Supplementary file 1 [file Table_1.docx]

Supplemental table 1: Shapiro-Wilk test of outcome data divided by group and pre and post intervention.

|  | Aerobic Group | | | | | | | |  | Stretching Group | | | | | | | |
| --- | --- | --- | --- | --- | --- | --- | --- | --- | --- | --- | --- | --- | --- | --- | --- | --- | --- |
|  | Fluid | | Crystal | | SR PedsQL | | PR PedsQL | |  | Fluid | | Crystal | | SR PedsQL | | PR PedsQL | |
|  | pre | post | pre | post | pre | post | pre | post |  | pre | post | pre | post | pre | post | pre | post |
| Shapiro-Wilk | 0.44 | 0.33 | 0.98 | 0.96 | 0.45236 | 0.04 | 0.8196 | 0.01 |  | 0.9349 | 0.2689 | 0.37 | 0.64 | 0.09 | 0.1 | 0.44 | 0.13 |

SR=self-report; PR=proxy-report
